# Supplementary material for: Placental genomics mediates genetic associations with complex health traits and disease
Source: Nat Commun. 2022 Feb 4;13:706. doi: 10.1038/s41467-022-28365-x (PMC8817049; doi:10.1038/s41467-022-28365-x)
Supplement: Supplementary file 1 — Supplementary Information [file 41467_2022_28365_MOESM1_ESM.pdf]

SUPPLEMENTAL INFORMATION

Genetic control of fetal placental genomics contributes to development of health and disease

SUPPLEMENTAL FIGURES

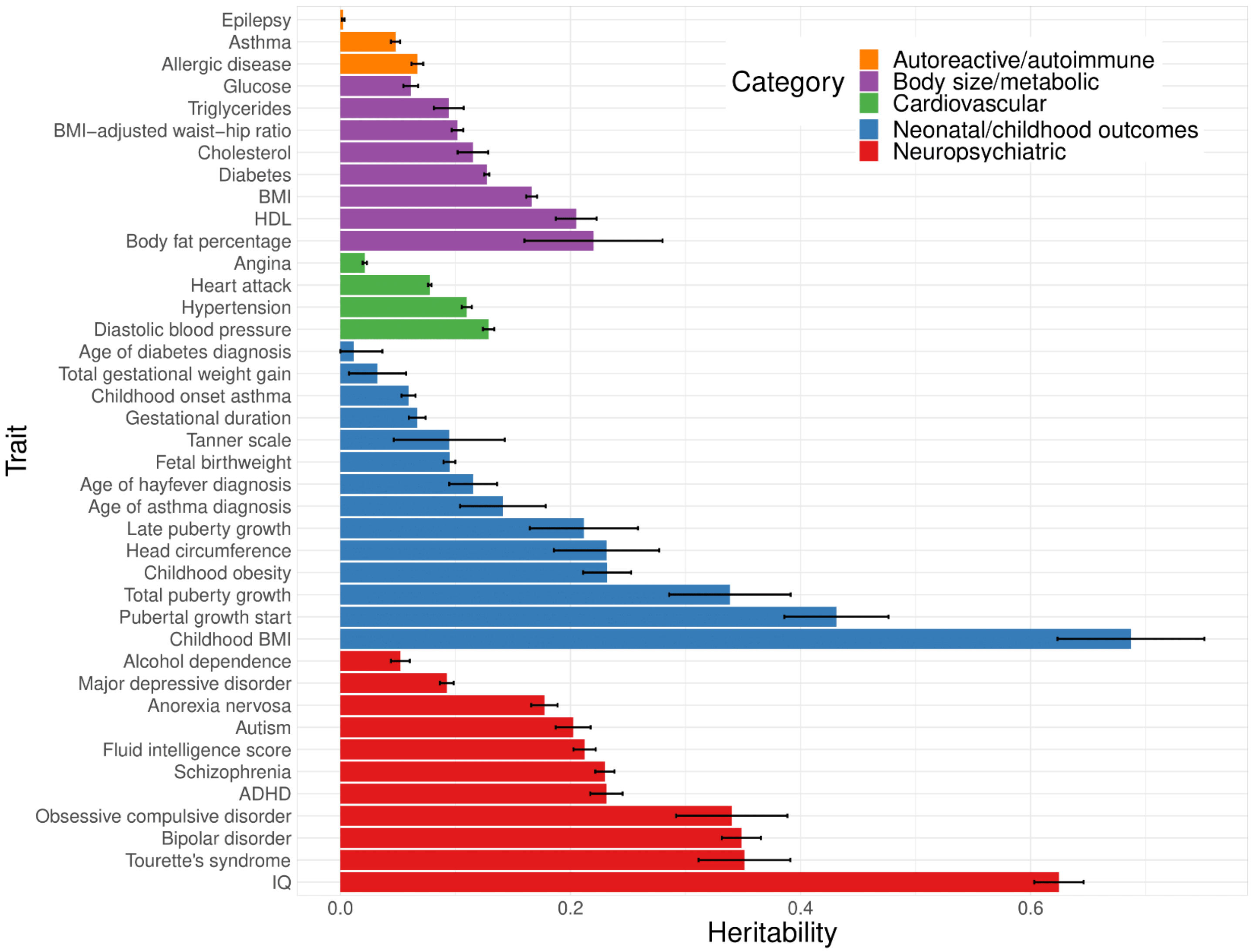

Figure S1: SNP heritability of 40 traits. Estimates of SNP heritability (magnitude of bar) with 95% Wald-type confidence interval (X-axis), grouped and colored by trait category (Y-axis). Sample sizes for trait are provided in Supplemental Table S1.

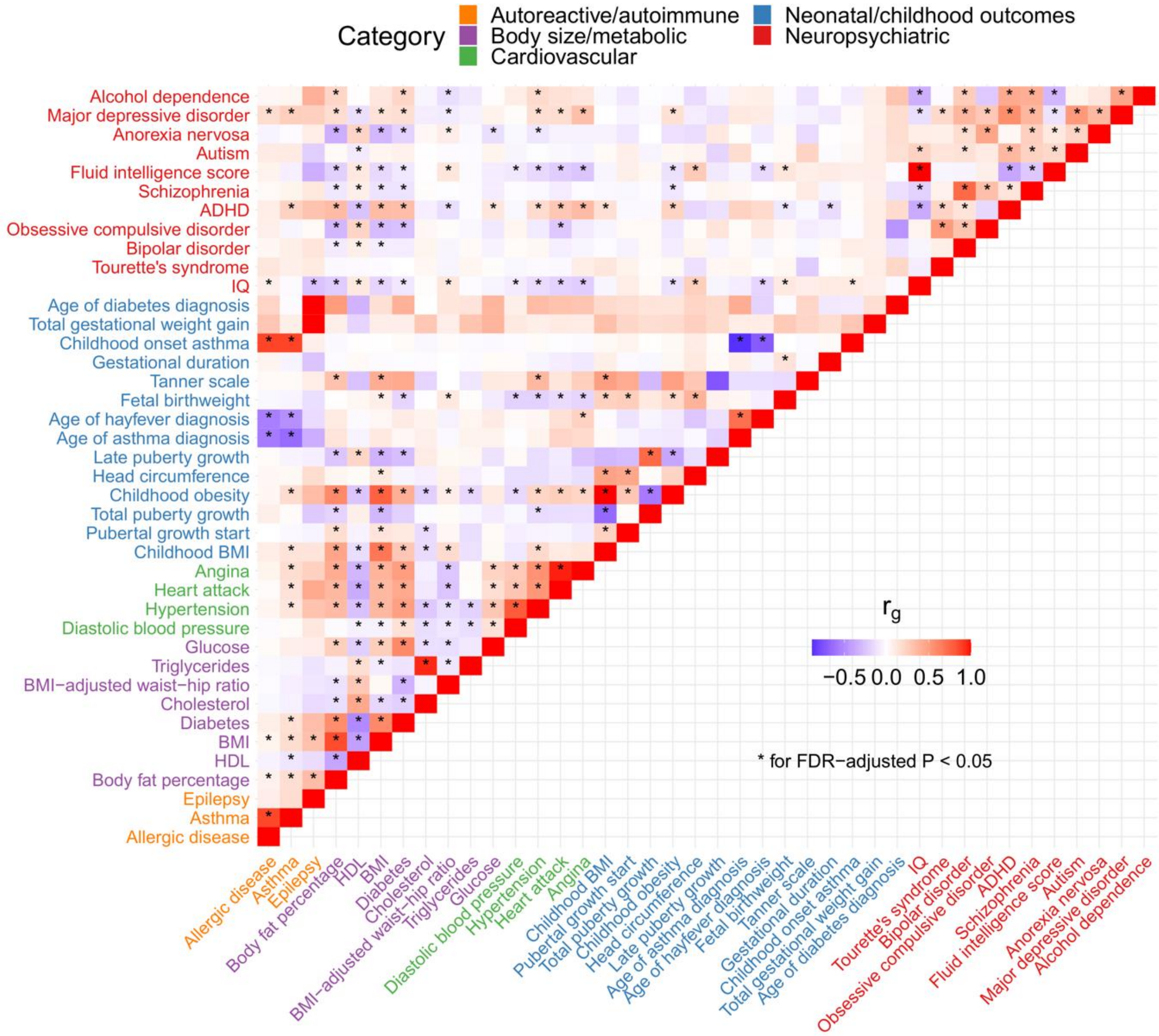

Figure S2: SNP-based genetic correlation between 40 traits. Heatmap of estimates of SNP-based genetic correlated between traits, grouped and colored by trait category. Correlations are marked with an asterisk are significantly non-zero with FDR-adjusted $P < 0.05$ (two-sided Wald-type test). Exact P-values are provided in Supplemental Table S3.

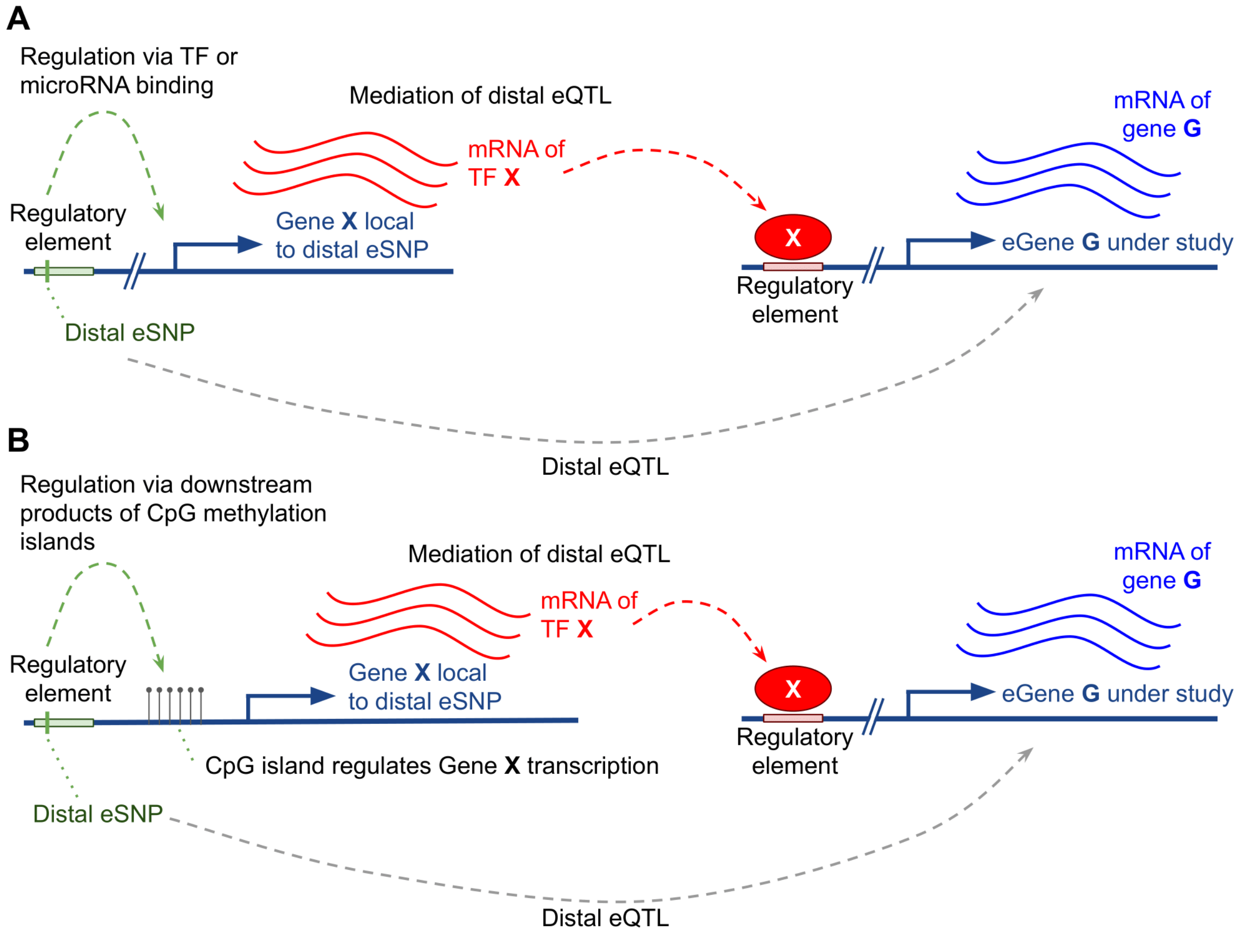

Figure S3: **Example of a biological mechanism MOSTWAS leverages in its predictive models**. Here, assume a SNP (in green) within a regulatory element affects the transcription of gene X (A) or the hyper- or hypomethylation of a CpG island upstream of gene X (B) that codes for a transcription factor or a microRNA hairpin. Transcription factor or microRNA X then binds to a distal regulatory region and affects the transcription of gene G. The association between the expression of gene X and gene G is leveraged in the first step of MeTWAS. A distal-eQTL association is also conferred between this distal-SNP and the eGene G, which is leveraged in the DePMA training process.

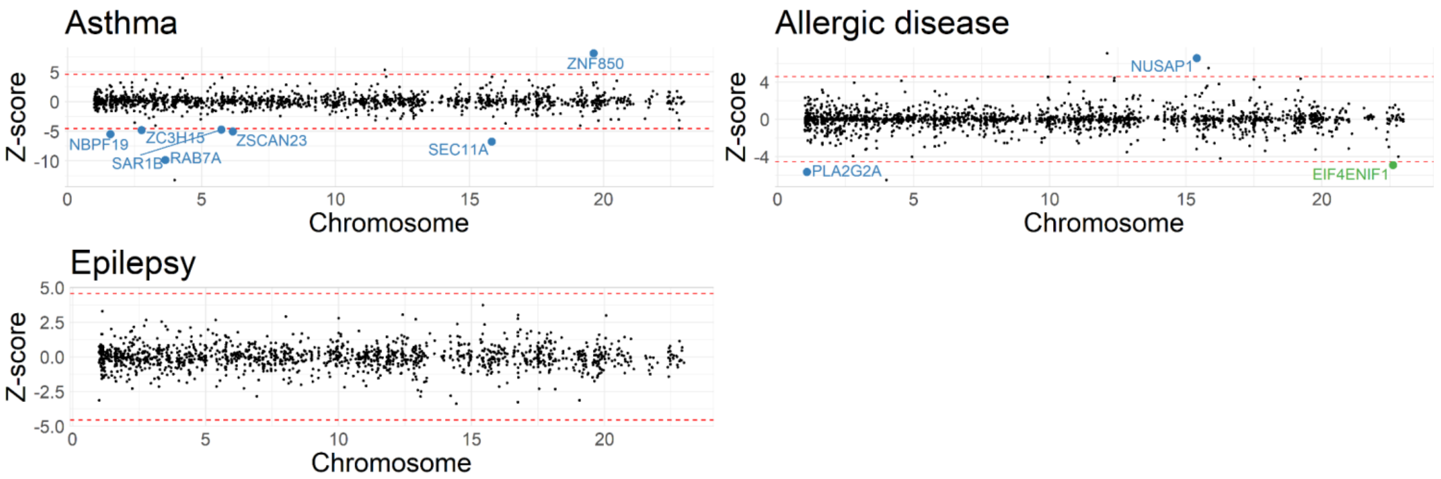

Figure S4: TWAS Miami plots for autoimmune/autoreactive disorders. Weighted two-sided Z-scores for TWAS associations (Y-axis) over genomic location of genes (X-axis). Red lines show Z-scores corresponding to P < 2.5×10^{-6}. Genes labelled have P < 2.5×10^{-6}, nominal permutation P < 0.05, and genes in green showed Benjamini-Hochberg FDR-adjusted P < 0.05 for the distal-SNPs added-last test.

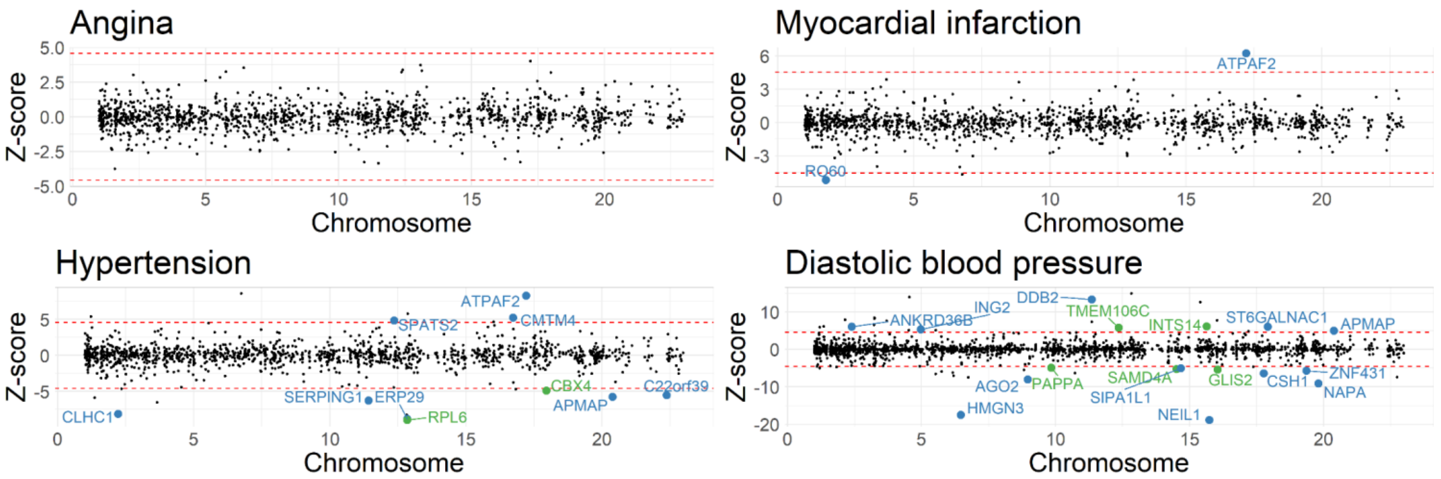

Figure S5: TWAS Miami plots for cardiovascular disorders. Weighted two-sided Z-scores for TWAS associations (Y-axis) over genomic location of genes (X-axis). Red lines show Z-scores corresponding to P < 2.5×10^{-6}. Genes labelled have P < 2.5×10^{-6}, nominal permutation P <0.05, and genes in green showed Benjamini-Hochberg FDR-adjusted P < 0.05 for the distal-SNPs added-last test.

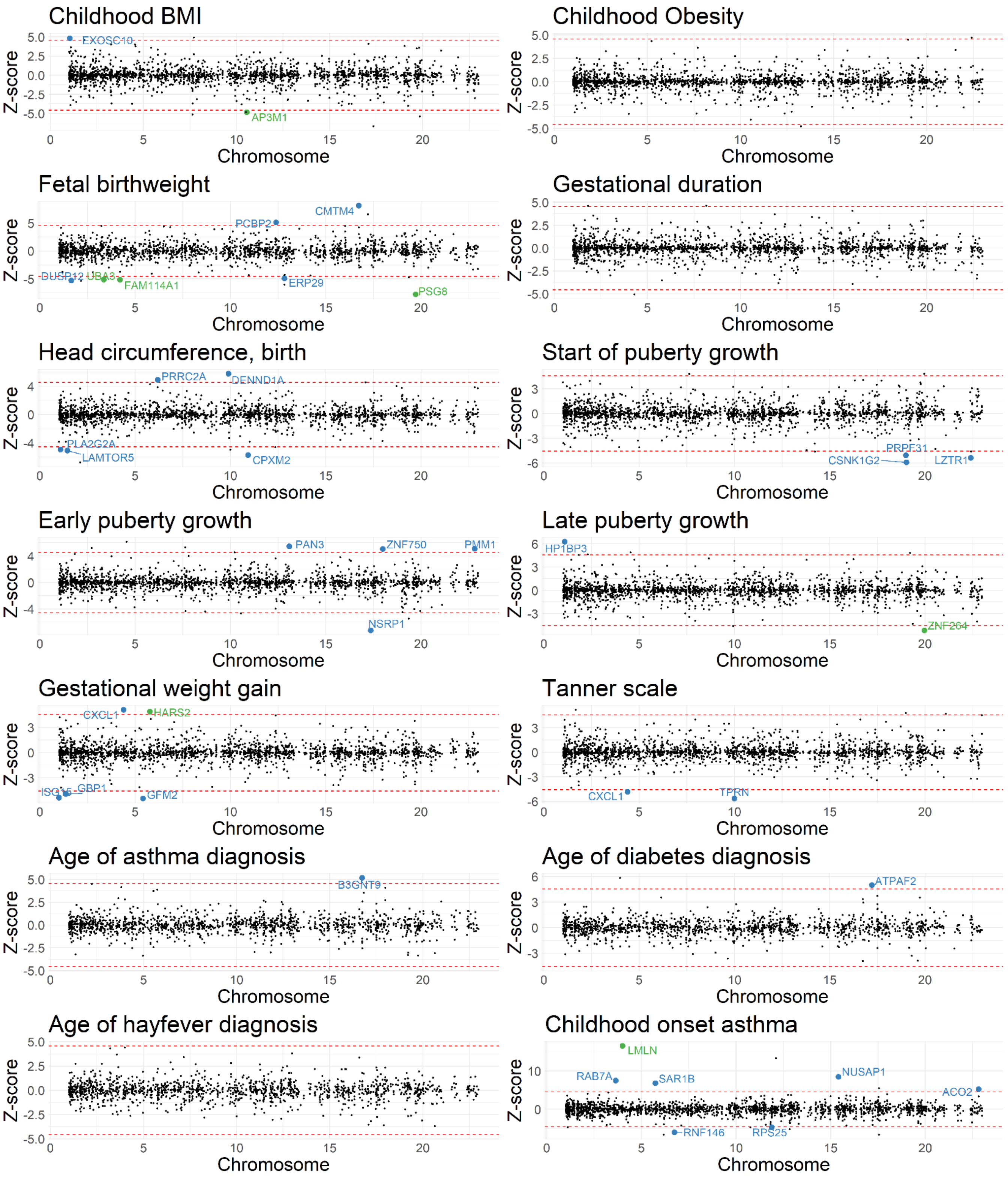

Figure S6: TWAS Miami plots for neonatal/childhood outcomes. Weighted two-sided Z-scores for TWAS associations (Y-axis) over genomic location of genes (X-axis). Red lines show Z-scores corresponding to P < 2.5×10^{-6}. Genes labelled have P < 2.5×10^{-6}, nominal permutation P < 0.05, and genes in green showed Benjamini-Hochberg FDR-adjusted P < 0.05 for the distal-SNPs added-last test.

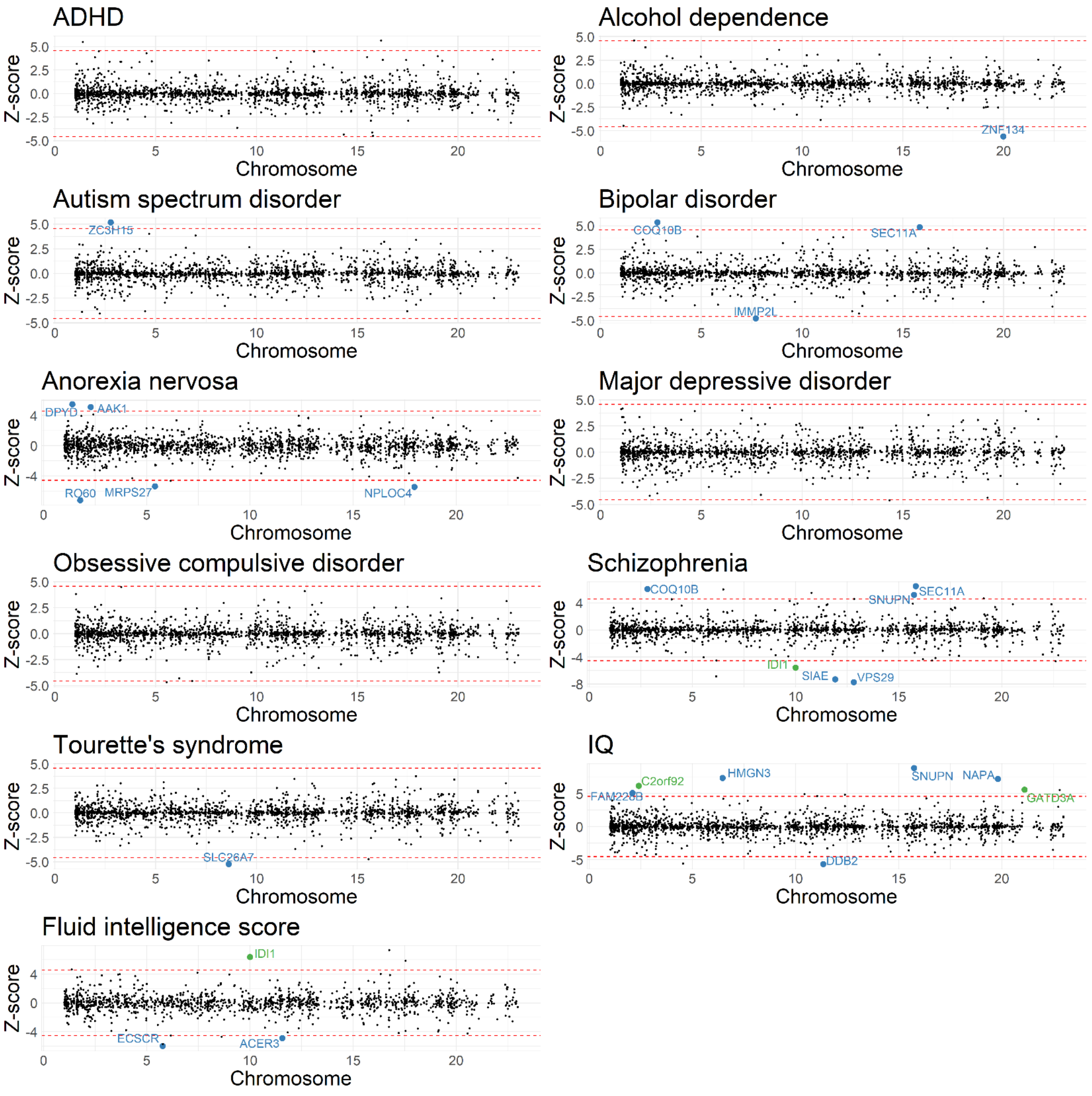

Figure S7: TWAS Miami plots for neuropsychiatric outcomes. Weighted two-sided Z-scores for TWAS associations (Y-axis) over genomic location of genes (X-axis). Red lines show Z-scores corresponding to $P < 2.5 \times 10^{-6}$. Genes labelled have $P < 2.5 \times 10^{-6}$, nominal permutation $P < 0.05$, and genes in green showed Benjamini-Hochberg FDR-adjusted $P < 0.05$ for the distal-SNPs added-last test.
.

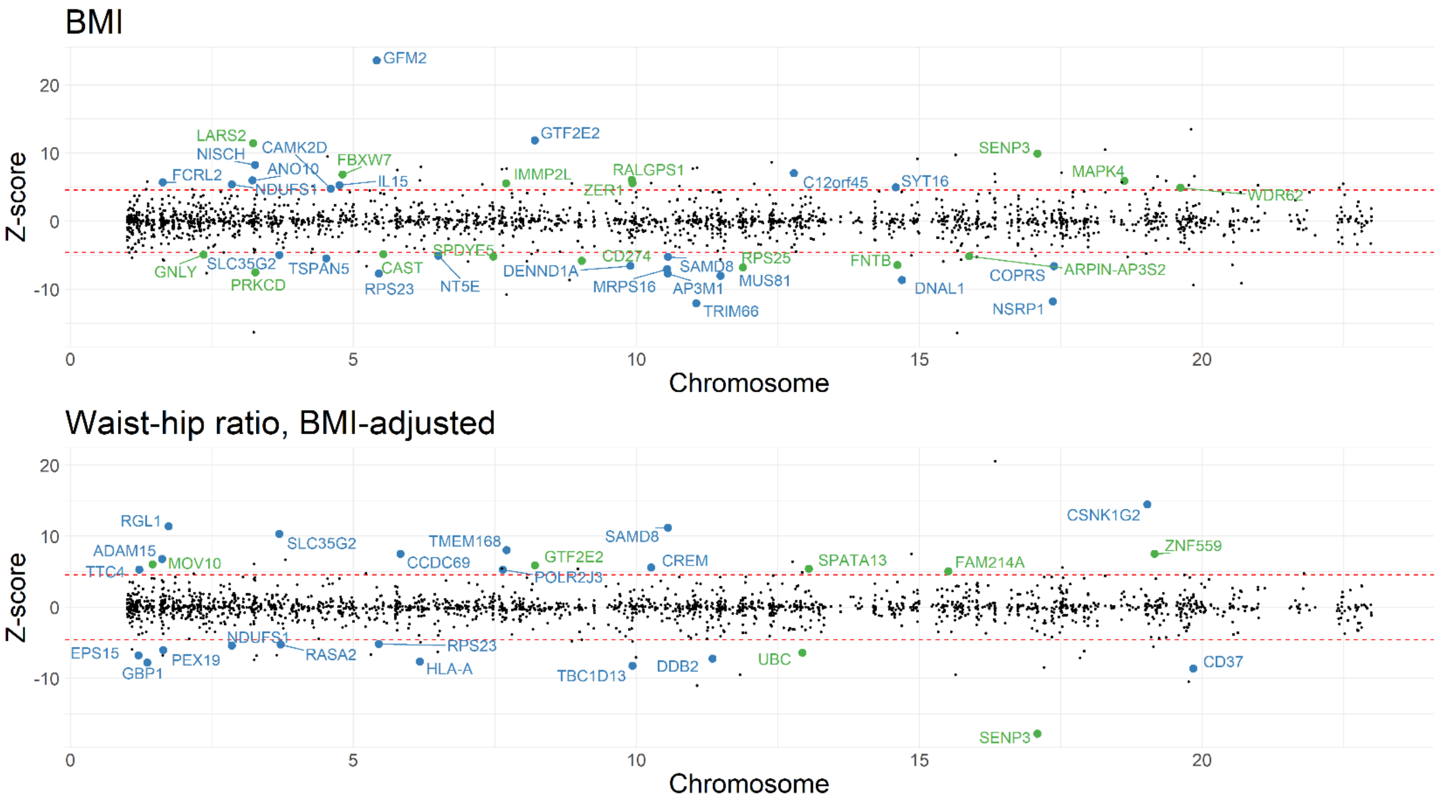

Figure S8: TWAS Miami plots for BMI and BMI-adjusted waist-hip ratio. Weighted two-sided Z-scores for TWAS associations (Y-axis) over genomic location of genes (X-axis). Red lines show Z-scores corresponding to $P < 2.5 \times 10^{-6}$. Genes labelled have $P < 2.5 \times 10^{-6}$, nominal permutation $P < 0.05$, and genes in green showed Benjamini-Hochberg FDR-adjusted $P < 0.05$ for the distal-SNPs added-last test.

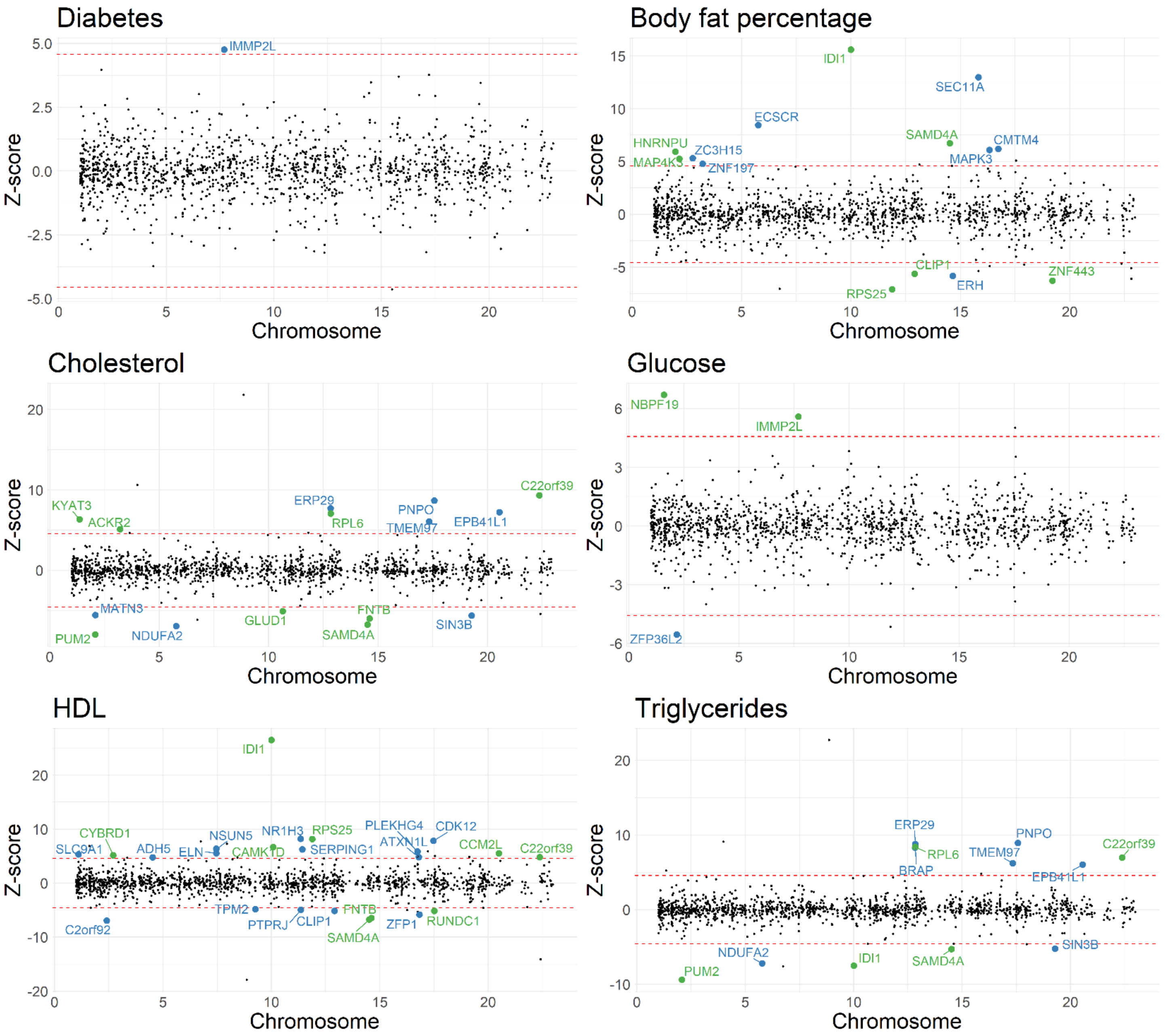

Figure S9: TWAS Miami plots for body size/metabolic traits, excluding BMI and BMI-adjusted waist-hip ratio. Weighted two-sided Z-scores for TWAS associations (Y-axis) over genomic location of genes (X-axis). Red lines show Z-scores corresponding to $P < 2.5 \times 10^{-6}$. Genes labelled have $P < 2.5 \times 10^{-6}$, nominal permutation $P < 0.05$, and genes in green showed Benjamini-Hochberg FDR-adjusted $P < 0.05$ for the distal-SNPs added-last test.

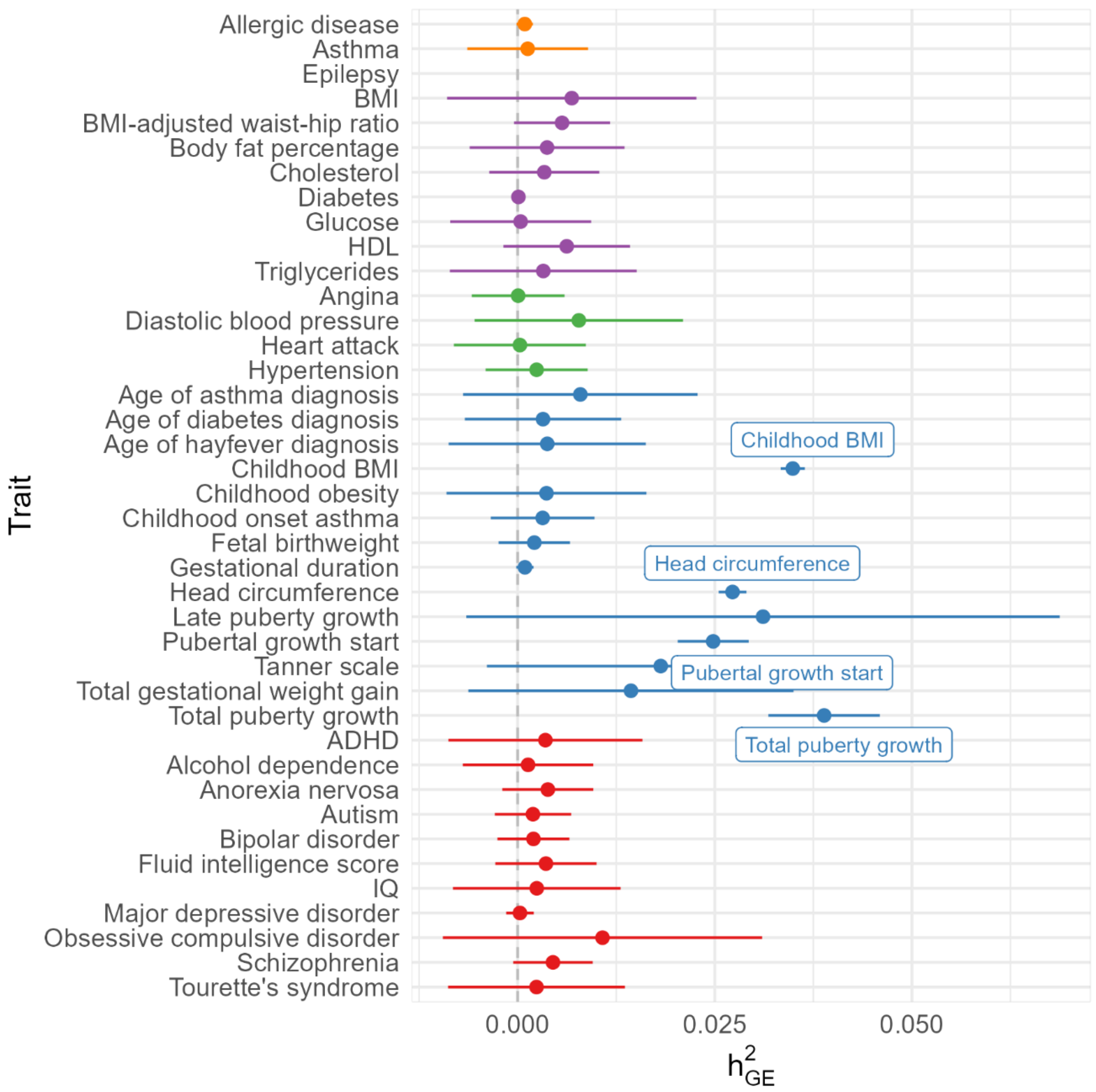

Figure S10: *Placental expression-mediated genetic heritability of traits.* Caterpillar plot of placental expression-mediated genetic heritability of traits (shown as points), colored by trait category. Wald-type 95% confidence intervals are provided for reference. Trait is labelled if the confidence interval does not intersect the null of $h^2_{GE} = 0$. Sample sizes are provided in Supplemental Table S1.

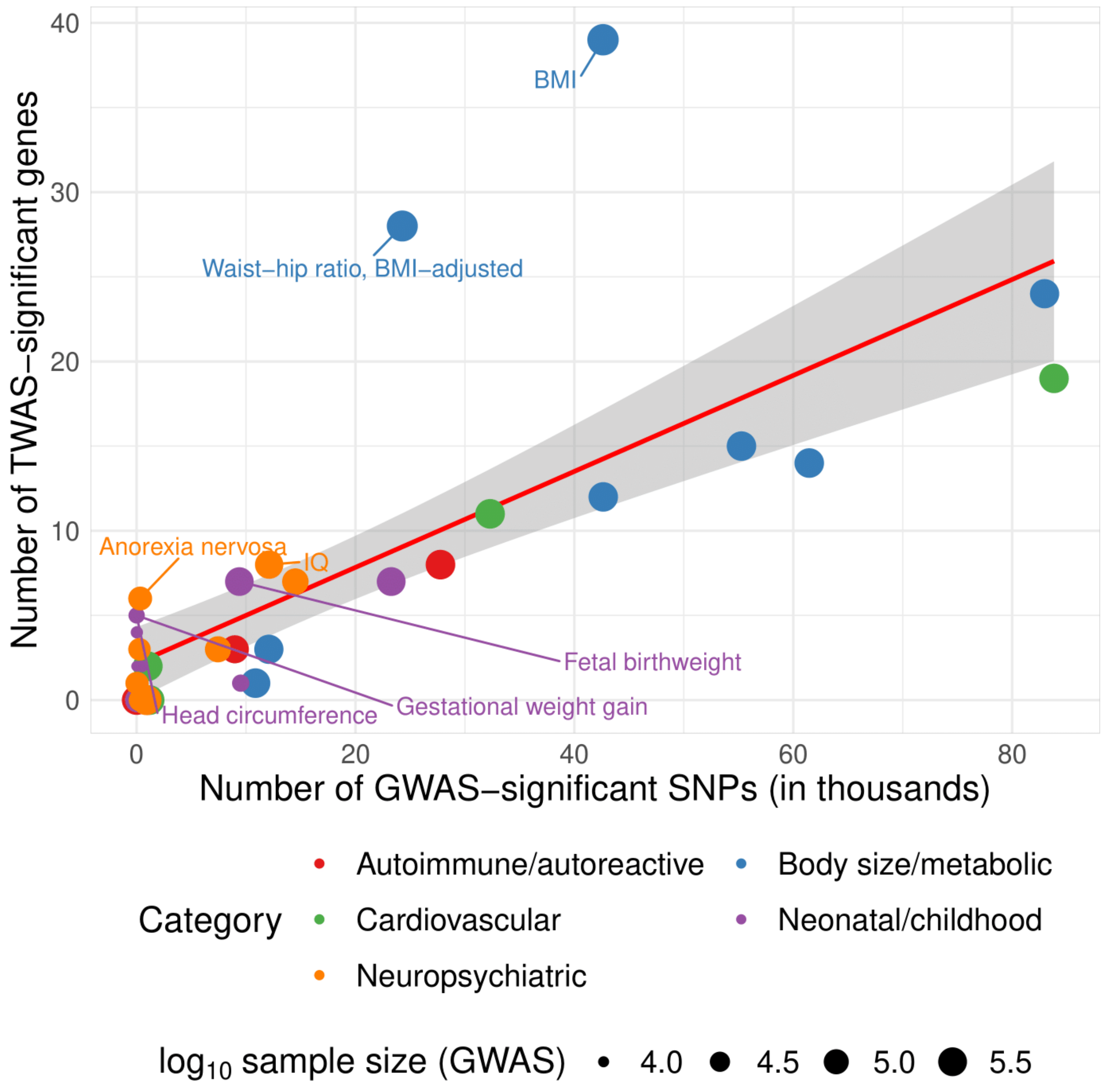

Figure S11: Comparison of GWAS and TWAS results across all 40 traits. Scatterplot of number of TWAS-significant genes (Y-axis) and number of GWAS-significant SNPs (X-axis) across all 40 traits, colored by category of the trait. The size of the point shows the $\log_{10}$ sample size of the GWAS. The red line and gray band provide a regression line and 95% Wald-type confidence band for the fitted values. Points are labelled if the point falls outside the confidence band.

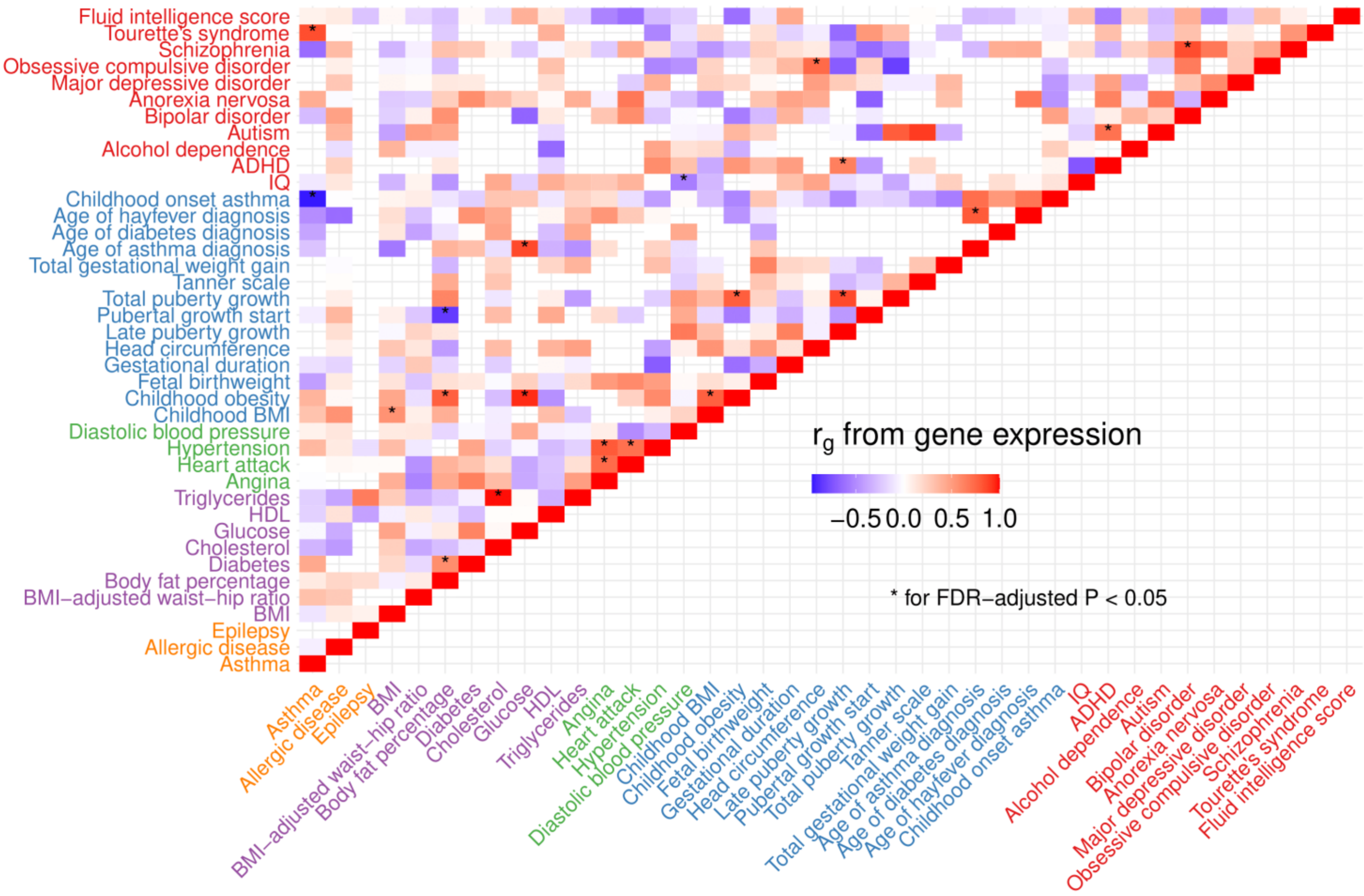

Figure S12: Heatmap of genetic correlations on the heritable gene expression level between 40 traits considered in TWAS analysis. Genetic correlations between traits at the level of the predicted expression of heritable genes. Correlations at FDR-adjusted P < 0.05 are marked with an asterisk (two-sided Wald-type tests). Autoimmune/autoreactive traits are colored in yellow, body size/metabolic in purple, cardiovascular in green, neonatal/childhood outcomes in blue, and neuropsychiatric in red. Exact P-values are provided in Supplemental Table S3.

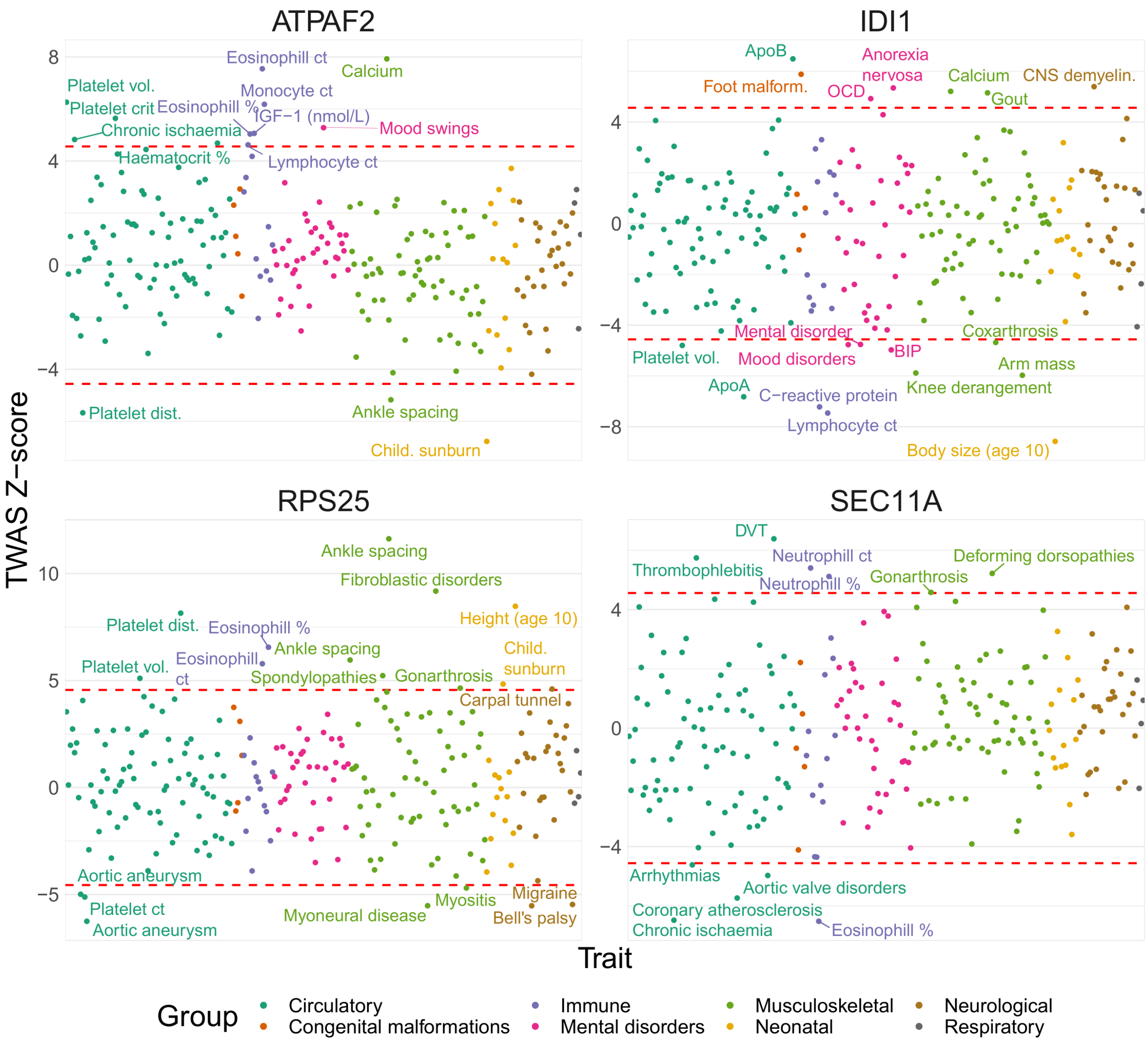

Figure S13: Miami plot of representative phenome-wide scans of GTAs in UKBB. Weighted burden Z-score (Y-axis) of GTA across all traits (X-axis), grouped and colored by ICD code block.

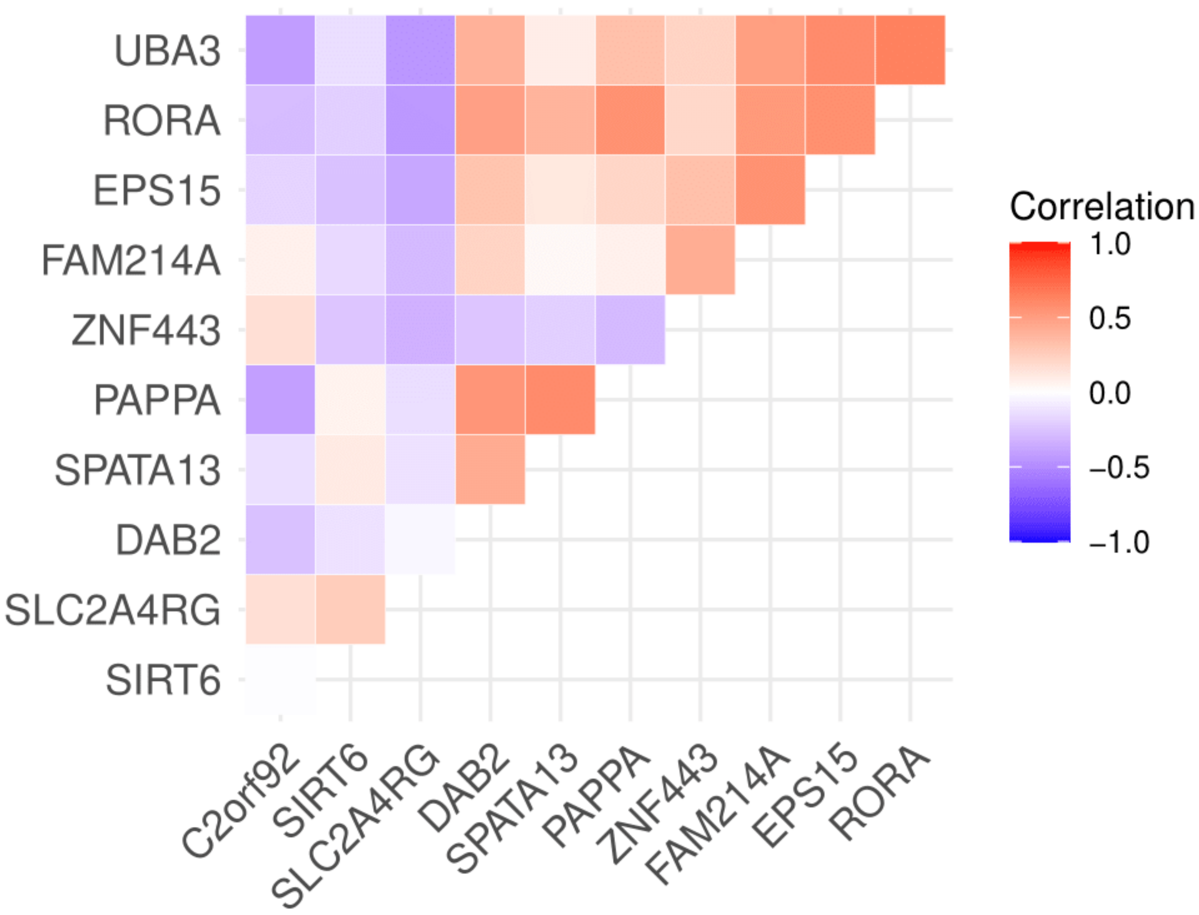

Figure S14: Heatmap of correlations between select regulatory protein-encoding and TWAS-identified genes in RICHS. Correlations between the RICHS expression of RPs (Y-axis) and associated TWAS genes identified by MOSTWAS in ELGAN (X-axis).

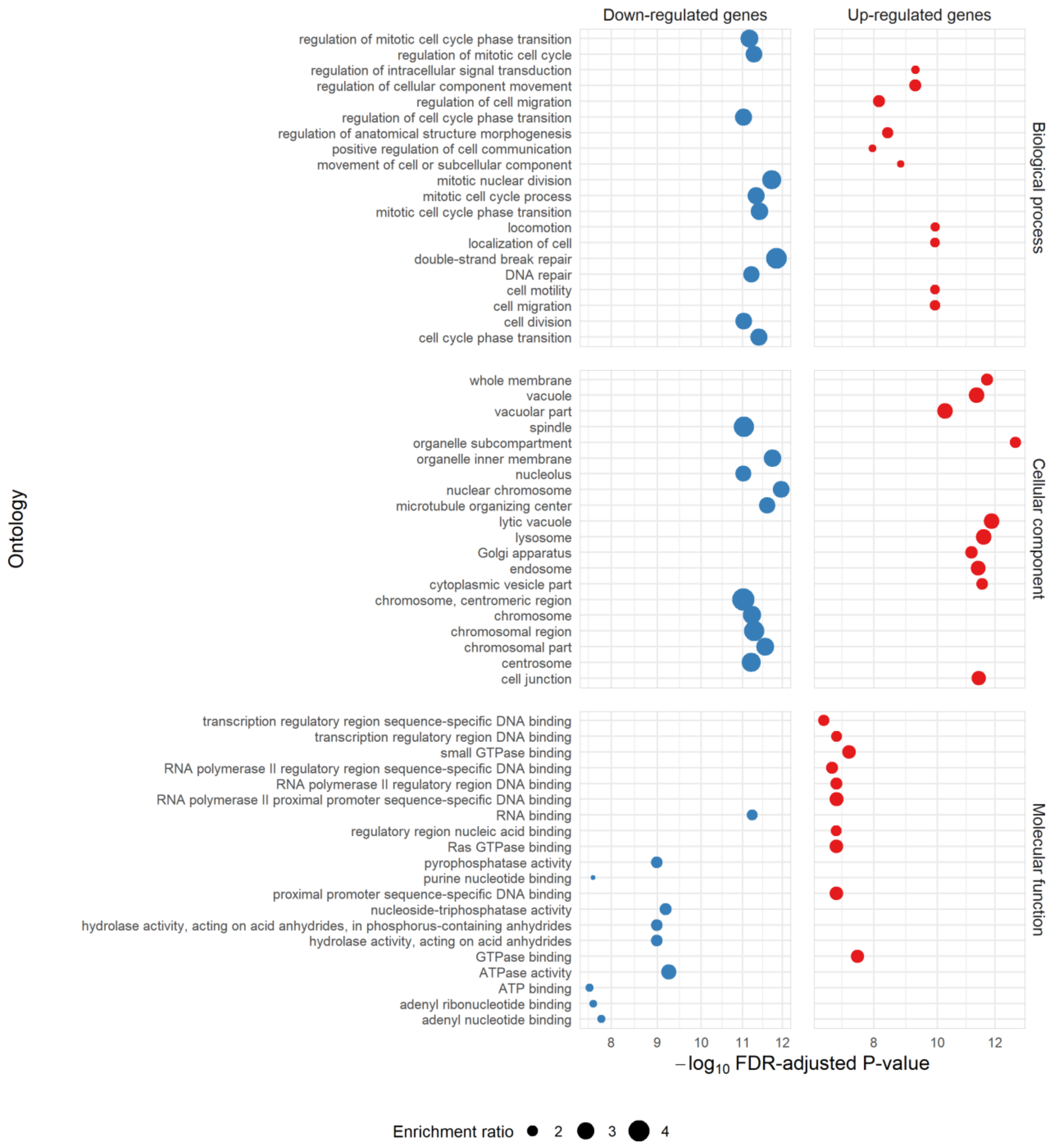

Figure S15: Over-representation enrichments of differentially expressed genes in *EPS15* knockdown. Enrichment plot of over-representation of biological process, cellular component, and molecular function ontologies (Y-axis) with -$\log_{10}$ FDR-adjusted P-value (X-axis; one-sided Fisher's exact test). The size of the point gives the relative enrichment ratio for the given pathway.
