# Supplementary material for: Placental genomics mediates genetic associations with complex health traits and disease
Source: Nat Commun. 2022 Feb 4;13:706. doi: 10.1038/s41467-022-28365-x (PMC8817049; doi:10.1038/s41467-022-28365-x)
Supplement: Supplementary file 3 — Description of Additional Supplementary Files [file 41467_2022_28365_MOESM3_ESM.pdf]

Description of Additional Supplementary Files

Supplementary Data 1: **Overview of 40 traits and GWAS consider in analysis**. The consortium, trait category, trait, URL for summary statistics, sample size, number of cases (if binary trait), SNP heritability estimate and standard error, Lambda GC, mean $\chi 2$ statistic, reference DOI for GWAS, and expression mediated heritability and standard errors (using all and all TWAS-significant genes) are provided in order.

Supplementary Data 2: Comparison of GWAS and TWAS associations. The category, trait, GWAS sample size, number of cases, number of significant GWAS SNPs (P < 5×10^{-8}), and number of significant total and GWAS-overlapping TWAS associations (P < 2.5×10^{-6}) are provided in order.

Supplementary Data 3: Genetic correlations between traits at SNP- and placenta-expression mediated levels. Genetic correlations, standard errors, two-sided Z-test statistic, P-value, FDR-adjusted P-value, and genetic covariance and standard errors are provided for all pairs of traits.

Supplementary Data 4: Demographic and clinical covariates summary statistics of ELGAN and RICHS samples.

Supplementary Data 5: Summary of in- and out-sample predictive performance of MOSTWAS placental expression models. Mean, standard deviation, 25% quantile, median, and 75% quantile of gene expression heritability, in-sample cross-validation R^2 in ELGAN, and out-sample R^2 in RICHS.

Supplementary Data 6: Summary of 248 significant TWAS gene-trait associations. For each gene and trait, the trait category, chromosomal position of the gene, expression heritability and associated likelihood ratio test P-value, cross-validation predictive performance for gene model, TWAS two-sided weighted burden Z-score and P-value, two-sided permutation test P-value, top SNP and P-value in GWAS among SNPs used in the gene model, distal Z-score and P-value, and identified mediators are provided, in order.

Supplementary Data 7: Over-representation analysis of TWAS genes. Biological process, molecular function, and PANTHER pathway ontologies enriched for TWAS-identified genes associated with each trait at FDR-adjusted P < 0.05.

Supplementary Data 8: Genetic correlations between traits at placental expression-mediated level. For each pair of traits, the genetic correlation, standard error, t-statistic and associated degrees of freedom and P-value is provided.

Supplementary Data 9: Results of fine-mapping of overlapped TWAS genes using FOCUS. Overlapping genes are provided, with the associated trait, chromosomal positions, TWAS two-sided weighted burden Z-scores and P-values, top GWAS SNP information, posterior inclusion probability, and whether they are included in the credible set for the region. The distal Z-score is also provided.

Supplementary Data 10: TWAS-significant genes that do not overlap significant GWAS loci (within 500 kB)

Supplementary Data 11: Susceptibility genes associated with multiple traits. TWAS gene, chromosomal location, and associated trait are provided with genetic correlations between traits at SNP level are provided if significant FDR-adjusted $P < 0.05$.

Supplementary Data 12: Results of ELGAN phenome-wide scan of neonatal outcomes. For each gene and ELGAN phenotype, the effect size, standard error, Wald-type adjusted 95% confidence interval, two-sided Z-score, P-value, and FDR-adjusted P-value are provided.

Supplementary Data 13: Cis-GReX correlations of TWAS-identified genes with metabolic traits in the Hybrid Mouse Diversity Panel. For each correlation at Spearman correlated test FDR-adjusted P < 0.10, the dataset, gene (mouse analog), trait, correlation, and P-value are provided.

Supplementary Data 14: Over-representation analysis of transcription factors identified as mediators. For the transcription-factor encoding genes identified as mediators, functional categories, ontologies, FDR-adjusted P-value of enrichment for one-sided Fisher's exact test, number of overlapping genes in the ontology, and the total number of genes in the ontology is given.

Supplementary Data 15: Trans-eQTL scan using GBAT in RICHS between genetic loci local to MOSTWAS-identified regulatory protein-encoding genes and the expression of the target TWAS gene. The effect size, P-value of two-sided Z-test, and FDR-adjusted P-value are provided.

Supplementary Data 16: Results from MR-Egger to assess causal effects of transcription factors on targeted TWAS genes. For each RP-TWAS pair, the causal estimate, confidence interval, P-value for two-sided Wald-type test, residual standard error, heterogeneity statistic, and heterogeneity P-value for heterogeneity Chi-squared test are provided.

Supplementary Data 17: MOSTWAS-identified CpG site mediators found within ENCODE-identified placenta cis-regulatory sites. For each CpG site mediator that overlaps with a placental cis-regulatory stie, the chromosomal location of the regulatory site, the classification of the regulatory site, tissue, gestational time, sex, and accession number are provided.

Supplementary Data 18: Summary statistics of down-regulated differentially expressed genes in EPS15 knockdown cells. For each gene with FDR-adjusted $P < 0.01$, we provide the gene name, $\log_2$ fold change, standard error, and P-value of two-sided Wald-type test.

Supplementary Data 19: Summary statistics of up-regulated differentially expressed genes in *EPS15* knockdown cells. For each gene with FDR-adjusted $P < 0.01$, we provide the gene name, $\log_2$ fold change, standard error, and P-value of two-sided Wald-type test.

Supplementary Data 20: Over-representation analysis of down-regulated genes. Biological process, molecular function, and PANTHER and KEGG pathway ontologies enriched for down-regulated genes in *EPS15* knockdown cells associated with each trait at FDR-adjusted P < 0.05 for one-sided Fisher's exact test.

Supplementary Data 21: Over-representation analysis of up-regulated genes. Biological process, molecular function, and PANTHER and KEGG pathway ontologies enriched for up-regulated genes in *EPS15* knockdown cells associated with each trait at FDR-adjusted P < 0.05 for one-sided Fisher's exact test.
