# Supplementary material for: Placental genomics mediates genetic associations with complex health traits and disease
Source: Nat Commun. 2022 Feb 4;13:706. doi: 10.1038/s41467-022-28365-x (PMC8817049; doi:10.1038/s41467-022-28365-x)
Supplement: Supplementary file 5 — Reporting Summary [file 41467_2022_28365_MOESM5_ESM.pdf]

nature portfolio

Corresponding author(s): Arjun Bhattacharya, Rebecca Fry, Hudson Santos

Last updated by author(s): Aug 3, 2021

Reporting Summary

Nature Portfolio wishes to improve the reproducibility of the work that we publish. This form provides structure for consistency and transparency in reporting. For further information on Nature Portfolio policies, see our Editorial Policies and the Editorial Policy Checklist.

Statistics

For all statistical analyses, confirm that the following items are present in the figure legend, table legend, main text, or Methods section.

n/a	Confirmed	
☐	☒	The exact sample size (*n*) for each experimental group/condition, given as a discrete number and unit of measurement
☐	☒	A statement on whether measurements were taken from distinct samples or whether the same sample was measured repeatedly
☐	☒	The statistical test(s) used AND whether they are one- or two-sided<br>*Only common tests should be described solely by name; describe more complex techniques in the Methods section.*
☐	☒	A description of all covariates tested
☐	☒	A description of any assumptions or corrections, such as tests of normality and adjustment for multiple comparisons
☐	☒	A full description of the statistical parameters including central tendency (e.g. means) or other basic estimates (e.g. regression coefficient) AND variation (e.g. standard deviation) or associated estimates of uncertainty (e.g. confidence intervals)
☐	☒	For null hypothesis testing, the test statistic (e.g. *F*, *t*, *r*) with confidence intervals, effect sizes, degrees of freedom and *P* value noted<br>*Give P values as exact values whenever suitable.*
☐	☒	For Bayesian analysis, information on the choice of priors and Markov chain Monte Carlo settings
☒	☐	For hierarchical and complex designs, identification of the appropriate level for tests and full reporting of outcomes
☐	☒	Estimates of effect sizes (e.g. Cohen's *d*, Pearson's *r*), indicating how they were calculated

Our web collection on statistics for biologists contains articles on many of the points above.

Software and code

Policy information about availability of computer code

Data collection	No software for used directly in this study to collect data.
Data analysis	We provide details for all software used for analysis in the code availability statement:<br><br>Sample scripts for analysis are provided at https://github.com/bhattacharya-a-bt/dohad_twas. The MOSTWAS software is accessible at https://bhattacharya-a-bt.github.io/MOSTWAS/articles/MOSTWAS_vignette.html. We use the following software in this study: PLINK v1.9 (https://zzz.bwh.harvard.edu/plink/), eagle v2.4.1 (https://alkesgroup.broadinstitute.org/Eagle/), minimac4 (https://genome.sph.umich.edu/wiki/Minimac4), Salmon 1.5.1 (https://salmon.readthedocs.io/en/latest/salmon.html), HTG EdgeSeq System (https://www.htgmolecular.com/systems/edgeseq), RUVSeq 3.12 (https://bioconductor.org/packages/release/bioc/html/RUVSeq.html), limma 3.12 (https://bioconductor.org/packages/release/bioc/html/limma.html), minfi 3.12 (https://bioconductor.org/packages/release/bioc/html/minfi.html), sva 3.40 (https://bioconductor.org/packages/release/bioc/html/sva.html), liftover 1.16.0 (https://www.bioconductor.org/packages/release/workflows/html/liftOver.html), ldsc 1.0.1 (https://github.com/bulik/ldsc), MOSTWAS 1.0.0 (https://github.com/bhattacharya-a-bt/MOSTWAS), MatrixEQTL 2.3 (https://cran.r-project.org/web/packages/MatrixEQTL/index.html), GCTA GREML-LDMS v1.93.1 (https://yanglab.westlake.edu.cn/software/gcta/#Overview), RHOGE 2018-02-28 (https://github.com/bogdanlab/RHOGE), maxprobes 0.0.1 (https://github.com/markgene/maxprobes), tximeta 3.14 (https://bioconductor.org/packages/release/bioc/html/tximeta.html), DESeq2 3.14 (https://bioconductor.org/packages/release/bioc/html/DESeq2.html), MendelianRandomization 0.5.1 (https://cran.r-project.org/web/packages/MendelianRandomization/index.html), and GBAT (https://github.com/xuanyao/GBAT).

For manuscripts utilizing custom algorithms or software that are central to the research but not yet described in published literature, software must be made available to editors and reviewers. We strongly encourage code deposition in a community repository (e.g. GitHub). See the Nature Portfolio guidelines for submitting code & software for further information.

Data

Policy information about availability of data

All manuscripts must include a data availability statement. This statement should provide the following information, where applicable:

- Accession codes, unique identifiers, or web links for publicly available datasets
- A description of any restrictions on data availability
- For clinical datasets or third party data, please ensure that the statement adheres to our policy

ELGAN mRNA, miRNA, and CpG methylation data can be accessed from the NCBI Gene Expression Omnibus GSE154829 (https://www.ncbi.nlm.nih.gov/geo/query/acc.cgi?acc=GSE154829) and GSE167885 (https://www.ncbi.nlm.nih.gov/geo/query/acc.cgi?acc=GSE167885). ELGAN genotype data is protected, as subjects are still enrolled in the study; any inquiries or data requests must be made to RCF and HPS. GWAS summary statistics can be accessed at the following links: UK Biobank (https://pan.ukbb.broadinstitute.org/downloads), GIANT consortium (https://portals.broadinstitute.org/collaboration/giant/index.php/GIANT_consortium_data_files), PGC (https://www.med.unc.edu/pgc/download-results/), EGG consortium (https://egg-consortium.org/), and CTG Lab (https://ctg.cncr.nl/documents/p1651/SavageJansen_IntMeta_sumstats.zip). The RICHS eQTL dataset can be accessed via dbGaP accession number phs001586.v1.p1 (https://www.ncbi.nlm.nih.gov/projects/gap/cgi-bin/study.cgi?study_id=phs001586.v1.p1). Placental epigenomic annotations from the ENCODE Project are available from https://www.encodeproject.org/, with specific accession numbers in Supplemental Table S17 (13 different accession numbers). All models and full TWAS results can be accessed at https://doi.org/10.5281/zenodo.4618036122. The RNA-seq data generated in placental JEG-3 cells are deposited in the NCBI GEO Database under accession code GSE185071 (https://www.ncbi.nlm.nih.gov/geo/query/acc.cgi?acc=GSE185071). Source data for figures are provided in the Supplemental Tables.

Field-specific reporting

Please select the one below that is the best fit for your research. If you are not sure, read the appropriate sections before making your selection.

[x] Life sciences   [ ] Behavioural & social sciences   [ ] Ecological, evolutionary & environmental sciences

For a reference copy of the document with all sections, see nature.com/documents/nr-reporting-summary-flat.pdf

Life sciences study design

All studies must disclose on these points even when the disclosure is negative.

Sample size	No calculations were conducted prior to analysis. For model training, we used 272 samples of placental multi-omic data from the ELGAN study, determined by the maximum sample size of individuals with multiomic fetal placenta and fetal genetic data. Sample sizes for external GWAS sample sizes were also pre-determined, as we reanalyze these GWAS presented in published manuscripts with TWAS. TWAS boosts power to detect gene-trait associations compared to GWAS, and our methods boosts TWAS power beyond traditional TWAS methods. Hence, our sample sizes are sufficiently large. For differential expression analysis for in-vitro studies, despite small sample sizes (4 samples per group), we use effective false discovery control processes: removing genes with low counts and high dispersions and using the Benjamini-Hochberg procedure.<br><br>For exploratory study of transcriptomic consequences of EPS15 knockdown in JEG-3 cells, 2 biological replicates (with 2 technical duplicates each) in control and knockdown groups were used. Sample sizes were maximized based on limited availability of reagents, and we restrict our results of differentially expressed genes to those are large fold-changes.
Data exclusions	No data was excluded from this study.
Replication	Expression models trained in the ELGAN study were validated in the Rhode Island Child Health Study to evaluate replicability of trans-eQTLs leveraged in the expression models. We have not conducted further in-vitro studies to confirm experimental results but our experiments are conducted in biological and technical duplicate.
Randomization	This is not relevant to our study. We use observational data for the transcriptome-wide association studies, and the in-vitro experiments were a controlled experiment using independently cultured cell lines.
Blinding	Blinding is not relevant to our study as we only use existing observational data and in-vitro experiments.

Reporting for specific materials, systems and methods

We require information from authors about some types of materials, experimental systems and methods used in many studies. Here, indicate whether each material, system or method listed is relevant to your study. If you are not sure if a list item applies to your research, read the appropriate section before selecting a response.

Materials & experimental systems

n/a	Involved in the study
☒	☐ Antibodies
☐	☒ Eukaryotic cell lines
☒	☐ Palaeontology and archaeology
☒	☐ Animals and other organisms
☐	☒ Human research participants
☒	☐ Clinical data
☒	☐ Dual use research of concern

Methods

n/a	Involved in the study
☒	☐ ChIP-seq
☒	☐ Flow cytometry
☒	☐ MRI-based neuroimaging

Eukaryotic cell lines

Policy information about cell lines

Cell line source(s)	The JEG-3 choriocarcinoma cells were purchased from the American Type Culture Collection (Manassas, VA; ATCC HTB-36).
Authentication	None of the cell lines used were authenticated.
Mycoplasma contamination	The cell lines were not tested for contamination.
Commonly misidentified lines (See ICLAC register)	TEC61 is listed as a mis-identified cell line that may be contaminated by JEG-3 cells. This information is not relevant, however, to this study as we use JEG-3 cells and not TEC61 cells.

Human research participants

Policy information about studies involving human research participants

Population characteristics	The Extremely Low Gestational Age Newborns (ELGAN) study (4UH3OD023348-03) is the source of data in the proposed study. The proposed study will include data from ELGAN children who currently range in age from 16 to 17 years. All these individuals must have participated in the three previous phases of the ELGAN Study (ages 2, 10, and 15 years). Approximately 52.5% of individuals are male. 71% of participants in this study identify as White and 23% identify as Black. Age of the individuals considered is not relevant, as multiomics data was collected at birth. Mean maternal age is 29.6 years. All relevant covariates are presented in Santos and Bhattacharya et al 2020, Molecular Autism (PMID: 33308293)
Recruitment	Individuals were recruited between April 2002 and August 2004, at 14 hospitals in five states in the U.S. All infants born at participating hospitals before 28 weeks of gestation were eligible. ELGAN enrolled 1,249 women giving birth before 28 weeks' gestation. Of 1,200 surviving ELGAN infants, 1,102 (92%) had clinical evaluations at age 2 years (ELGAN-1 Study). For a second clinical evaluation at age 10 years, ELGAN recruited a subset (n=966) of survivors for whom we had multiple outcome measurements and biospecimens (the ELGAN-2 Study); 889 (92%) returned for follow-up and were evaluated. Recently, at age 15 years (ELGAN-ECHO), 810 children (91.1% of those evaluated at age 10) completed a neurodevelopmental test battery and robust positive child health assessment. This proposal aligns with the scheduled ELGAN follow-up at 17 years of age to collect neurodevelopmental, including cognitive function, data.
Ethics oversight	Local IRBs (IRB #16-2535) approved this study.

Note that full information on the approval of the study protocol must also be provided in the manuscript.
